# Assessing the Image Quality of Digitally Reconstructed Radiographs from Chest CT

**DOI:** 10.1007/s10278-025-01406-9

**Published:** 2025-01-27

**Authors:** Olivier T. Paalvast, Omar Hertgers, Merlijn Sevenster, Hildo J. Lamb

**Affiliations:** 1 Leiden University Medical Center (LUMC), Leiden, the Netherlands; 2 Royal Philips B.V., Amsterdam, the Netherlands

**Keywords:** Artificial intelligence, Digitally reconstructed radiographs, Ultra-low-dose CT, Chest imaging, Image quality

## Abstract

Rising computed tomography (CT) workloads require more efficient image interpretation methods. Digitally reconstructed radiographs (DRRs), generated from CT data, may enhance workflow efficiency by enabling faster radiological assessments. Various techniques exist for generating DRRs. We conducted a pilot study to explore which DRR technique best approaches the image quality of chest X-Rays (CXR). We quantitatively and qualitatively compared four DRR techniques. A retrospective convenience sample of 217 patients who underwent both ultra-low-dose (ULD) chest CT and CXR was used. Four DRRs were generated per ULDCT, and CheXNet, a neural network trained to detect 14 diseases, was applied to CXRs and DRRs to compute area under the curve (AUC) scores. For qualitative assessment, six radiologists rated the image quality of the four DRRs generated from six ULDCTs on a Likert scale from 1 to 6 (‘not diagnostic quality’ to ‘diagnostic quality’) and provided feedback, which was analysed using inductive category development. CheXNet’s AUC for CXRs was 0.80, while DRR techniques ranged from 0.75 to 0.82 (*p* > 0.26). Radiologists rated the diagnostic quality of the DRRs between 3.0 and 3.5 on average. The SoftMip technique scored highest in both the quantitative (AUC = 0.82) and the qualitative (score = 3.5) evaluation. DRRs showed comparable disease detection performance to CXRs, suggesting non-inferiority. However, radiologists expressed concerns about DRR image quality, particularly in terms of resolution, noise, and overall look-and-feel. Addressing these limitations with advanced techniques may further align DRRs with the diagnostic standards of CXRs.

## Introduction

The number of CT examinations continues to rise annually, placing increasing demands on radiology departments to manage larger workloads efficiently [1]. As a result, there is a growing need for more streamlined image interpretation methods. Digitally reconstructed radiographs (DRRs), generated from CT scans, may enhance image reading by enabling faster assessment.

A DRR is a 2D projection that is generated by simulating X-ray paths through volumetric CT data [2–5]. Using standard computation techniques, DRRs can be efficiently produced from various viewing angles. These versatile images may support radiologists in their clinical diagnostic workflow. When derived from chest CT data, the resulting DRRs cover the same anatomical plane and viewing angles as a conventional chest X-ray (CXR).

In the literature, four different techniques are described for generating a DRR from CT data. Of these techniques, two focus on diagnostic chest applications [6, 7], one on simulating second-order effects like scatter and noise in DRRs in general [4], and one on ultra-low-dose (ULD) CT images in general [8]. Each technique is optimised for its respective application, resulting in four distinct DRRs that can be generated from any CT data. In this study, these four techniques are implemented to facilitate a comparison with conventional CXRs.

The aim of this pilot assessment is to evaluate the image quality of the four DRR techniques to determine which most closely approaches the diagnostic quality of CXRs. This evaluation consists of an objective quantitative approach using automatic pathology detection and a subjective qualitative approach with radiologists rating DRRs. A convenience sample of same-day ULDCT chest CT scans and CXRs in 217 patients is utilised. Unlike previous studies that primarily focused on DRRs derived from standard-dose CT scans, this study uniquely compares multiple DRR techniques using ULDCT data, which delivers radiation exposure comparable to CXRs. Additionally, the inclusion of radiologist-led qualitative assessments provides new insights into the clinical perception of DRR image quality, expanding on earlier machine learning-focused evaluations.

## Methods

The Institutional Review Board waived patient consent for use of a retrospective dataset (number NL20210610001). The dataset that is used to generate the DRRs originates from the Leiden University Medical Centre hospital and was originally collected to assess the differences between a CXR and an ULDCT for patient management [9]. All participants in the qualitative evaluation provided written informed consent with regard to their participation and the sharing of aggregate personal information.

### Creating Digitally Reconstructed Radiographs

**Fig. 1:**
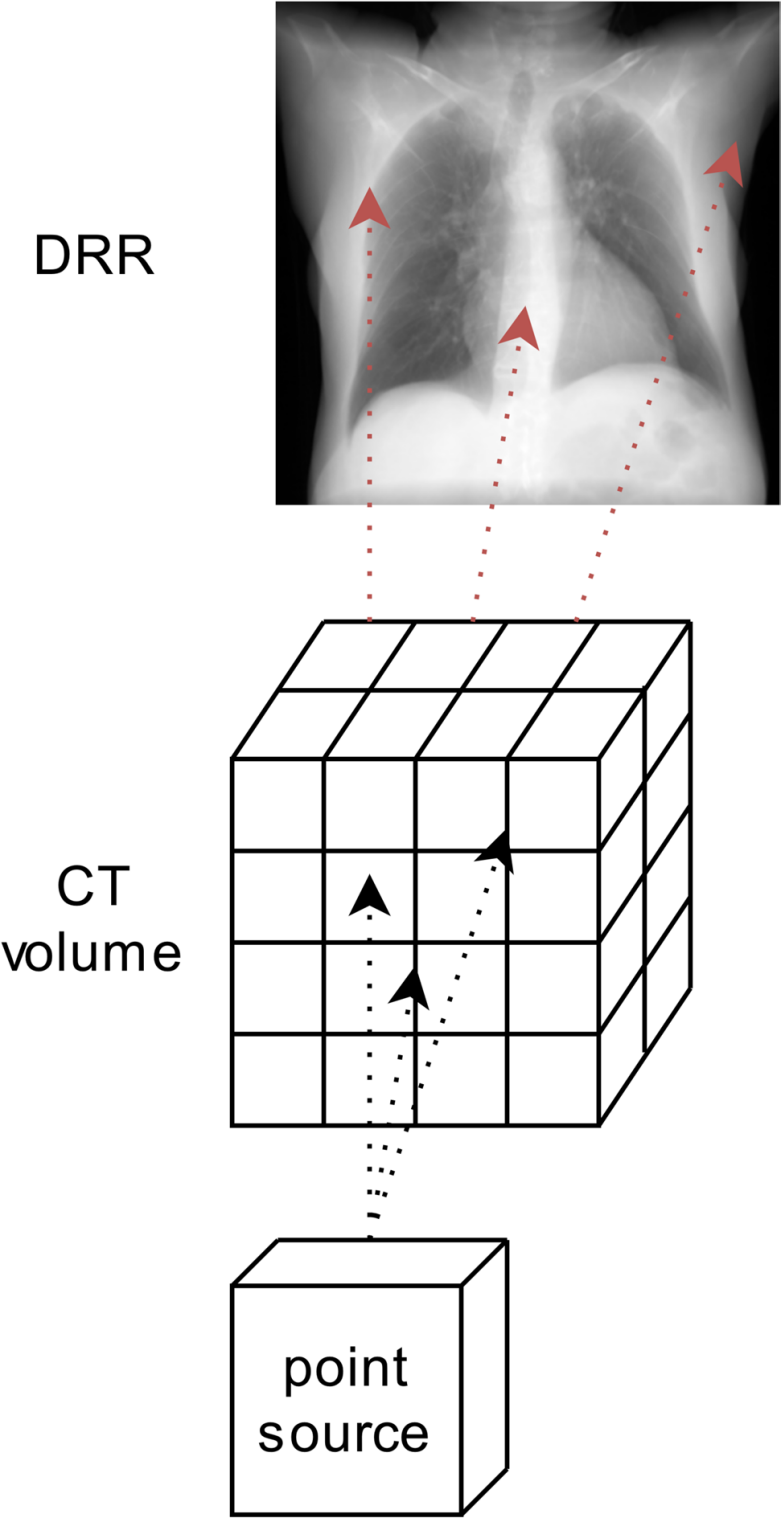
Schematic representation of a point-source-based DRR. Virtual X-rays are traced from the point-source to the detector. Because of the point source nature, a certain level of divergence is seen in this DRR

**Fig. 2:**
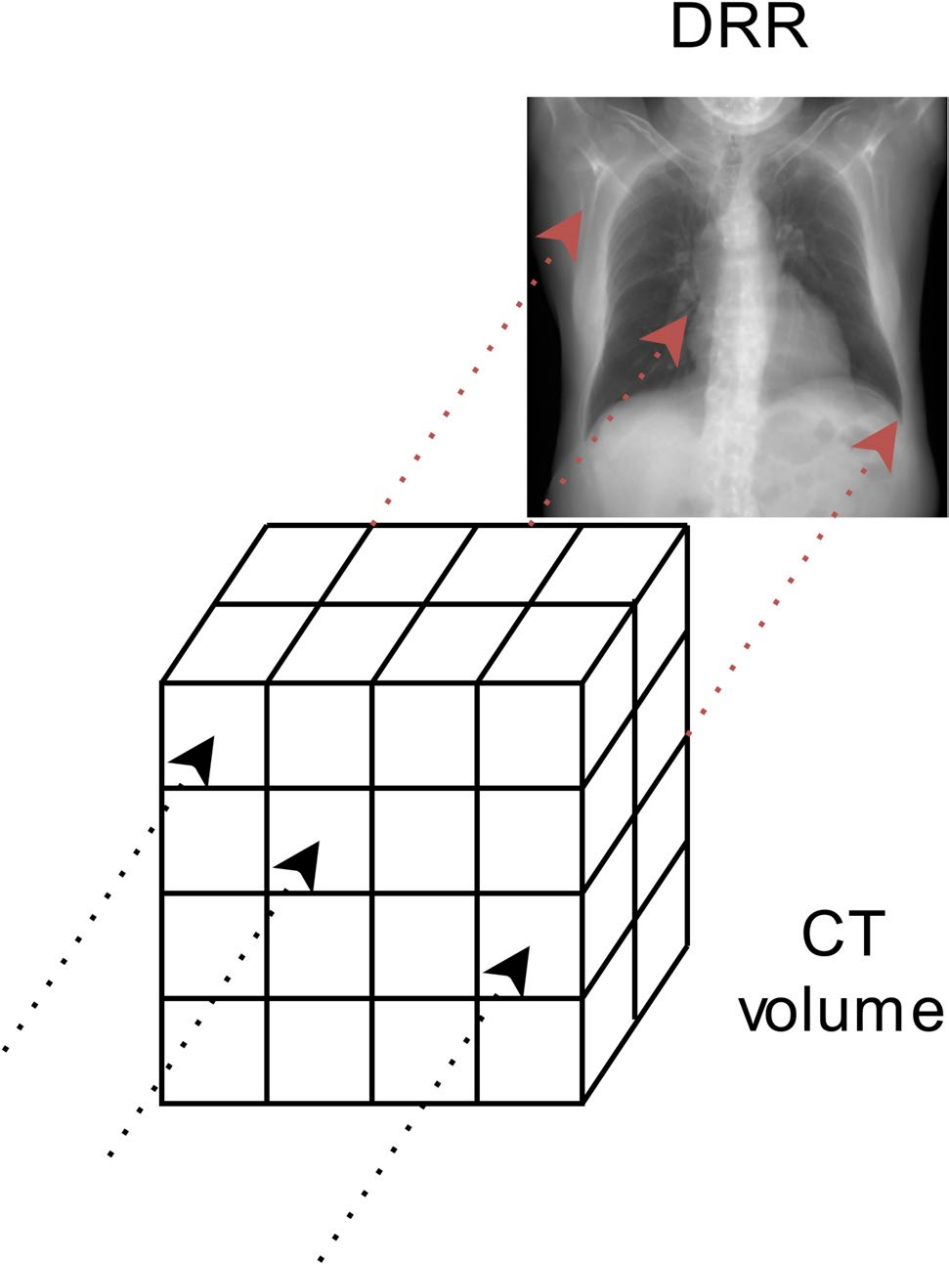
Schematic representation of a parallel based DRR. The virtual X-ray only interacts with voxels in a parallel straight line running from the front to the back of the volume. No divergence is present in the resulting DRR

X-rays may be traced through CT volumes using two primary methods: (1) point-source-based projection and (2) parallel-based projection. The point-source-based projection, illustrated in Fig. 1, closely mimics the clinical method of obtaining CXRs, and introduces a slight divergence to the generated DRR. In contrast, the parallel-based projection, depicted in Fig. 2, differs by eliminating divergence, as projection is calculated using parallel and consecutive voxels. Given the subject is positioned at a considerable distance to the X-ray source, the difference between these projections is minimal, manifesting primarily as a slight divergence in the point-source projections. The technique used to calculate the attenuation of the traced virtual X-rays plays a significant role in determining the image output.

We identified four distinct DRR generation techniques from the literature, comprising one point-source-based projection and three parallel-based projections. The point-source-based projection, developed by Unberath et al. [4], calculates attenuation using material decomposition by assigning a tissue-based weighing function to the interacting virtual X-rays and incorporating noise through scatter estimation.

The parallel-beam techniques differ primarily in voxel weighting long the line integral. The approach by Campo et al. [6] is characterised by the application of the Lambert–Beer law [10] to compute the attenuation of the virtual X-rays. Meyer et al. [8] modified this approach by introducing a sorting mechanism for all interacting voxels, with voxel weighting determined by the following relationship:

$${f}_{\mathrm{weight}}^{{\mathrm{Soft}}{\mathrm{Mip}}}\left(x\right)=\left\{\begin{array}{l}0.5x, x\le 50\\ 1.5x-0.5, {\mathrm{o}}{\mathrm{t}}{\mathrm{h}}{\mathrm{e}}{\mathrm{r}}{\mathrm{w}}{\mathrm{i}}{\mathrm{s}}{\mathrm{e}}\end{array}\right.$$

where $x$ represents the absolute position between of the voxel in the sorted array, ranging from 0 to 100. Carey et al. [7] described a similar technique involving voxel sorting and weighting. In their method, tomographic slabs were constructed based on a custom voxel weighting factor, optimised visually, resulting in a ‘wedge’ that defined the weighting factor for each voxel position within the sorted array.

To facilitate comparison with conventional CXRs, all DRRs are generated as posteroanterior (PA) projections of the CT data, although DRRs can be constructed for any desired viewing angle.

### Dataset

Data used in this study was gathered between January 2016 and December 2017. Each case in the study comprises a PA CXR and an ULDCT scan. All included CXR and ULDCT cases were performed on the same day, on the same patients, and for the same clinical indication to ensure consistency and minimize variability. Every case was systematically examined for pathology, cross-referenced with the corresponding clinical report, and subsequently annotated using the disease labels established in the CheXNet CXR classification model by Rajpurkar et al. [11]. The disease annotation was performed by two independent reviewers, both board-certified radiologists with subspecialty expertise in thoracic imaging and a minimum of 5 years of experience, who resolved discrepancies on a consensus basis using the CXR and the associated clinical report for the respective case.

ULDCT was selected as the source data for DRR generation to align with the study’s objective of examining the diagnostic utility of DRRs in a context where radiation exposure is minimal. This choice reflects a clinical scenario where DRRs could replace CXRs while adhering to low-dose imaging standards. Standard-dose CTs were not used to avoid confounding results with higher radiation exposure.

### Quantitative Evaluation

To quantitatively evaluate the different DRR techniques, we employed the CheXNet CXR disease classification network by Rajpurkar et al. [11]. The state-of-the-art CheXNet network provides an output of 14 disease class labels for a given CXR input image, corresponding to the disease class labels in the National Institute of Health dataset which it was trained on. Using the radiological reports as ground truth, we computed area under curve (AUC) scores for the CXRs and the DRRs generated for the corresponding ULDCTs.

Prior to analysis, input CXRs and DRRs were resized to a 224 × 224 image size using bicubic interpolation. The resulting AUCs for the disease classification performance were then compared for statistical significance using the DeLong [12] test.

### Qualitative Evaluation

To assess the potential clinical relevance of the four DRR construction methods, we conducted a qualitative evaluation with radiologists. The qualitative evaluation was performed using the DICOM viewer MicroDicom (version 2022.2) and was displayed on a 4K resolution display, representative of the screens commonly used in clinical practice. We recruited six radiologists with an average of 9 years of clinical experience reading CXRs to participate in the qualitative evaluation, where they assessed DRRs independently without viewing the corresponding CXRs to focus solely on the image quality of DRRs.

In this evaluation, participants were presented with six patient cases with known absence of pathology. The participants were informed of this, and the cases were presented in a randomised order. Participants assessed the diagnostic quality of the DRRs based on four anatomical regions: soft tissue, ossal structures, mediastinum, and lungs. For each case, they provided an overall diagnostic score and verbal feedback on the usability of the images. Diagnostic quality was rated using a 6-point Likert scale, where 1 corresponded to ‘not diagnostic quality’ and 6 corresponded to ‘diagnostic quality’. The questions were designed to address the relevant anatomical regions typically assessed on a CXR. The ratings were analysed for statistical significance using paired *t*-tests. The provided qualitative feedback was further analysed using inductive category development, as outlined by Mayring et al. [13].

## Results

**Fig. 3:**
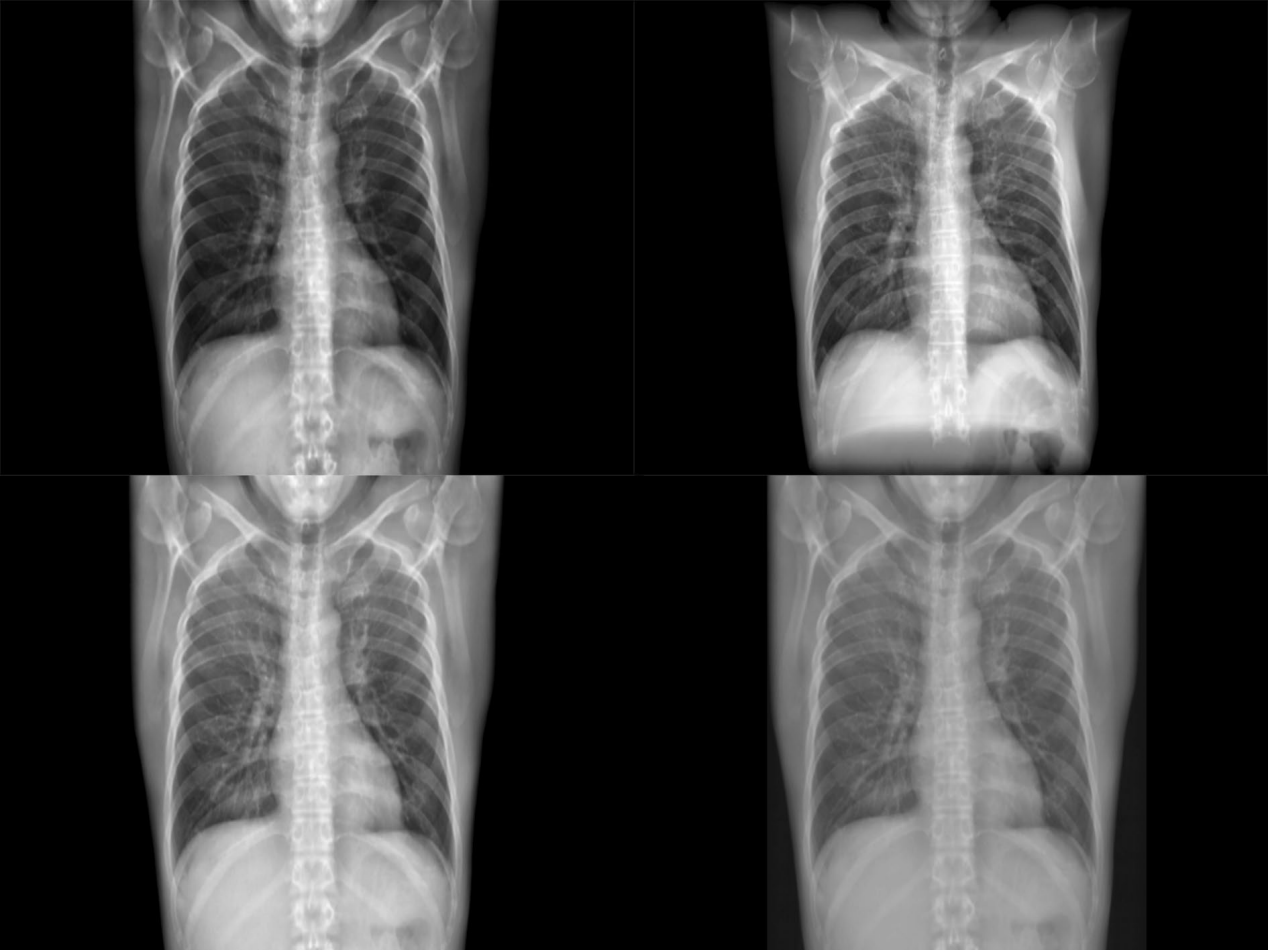
Four example DRRs generated from a single ULDCT case. In clockwise order starting on the top-right these are the DeepDRR (point-source) approach and the Campo, Carey, and Meyer parallel-projection approaches, respectively

All four DRR generation techniques were implemented successfully. For illustration purposes, an example of a DRR generated using each of the four methods for a single case is included in Fig. 3.

### Dataset

Of the 217 included cases, 20 cases were deemed unusable due to the absence of images (*n* = 8), the absence of radiological reports (*n* = 6), or incomplete presence of images (*n* = 6). The resulting 197 cases were used for the quantitative evaluation.

### Quantitative Evaluation

**Fig. 4:**
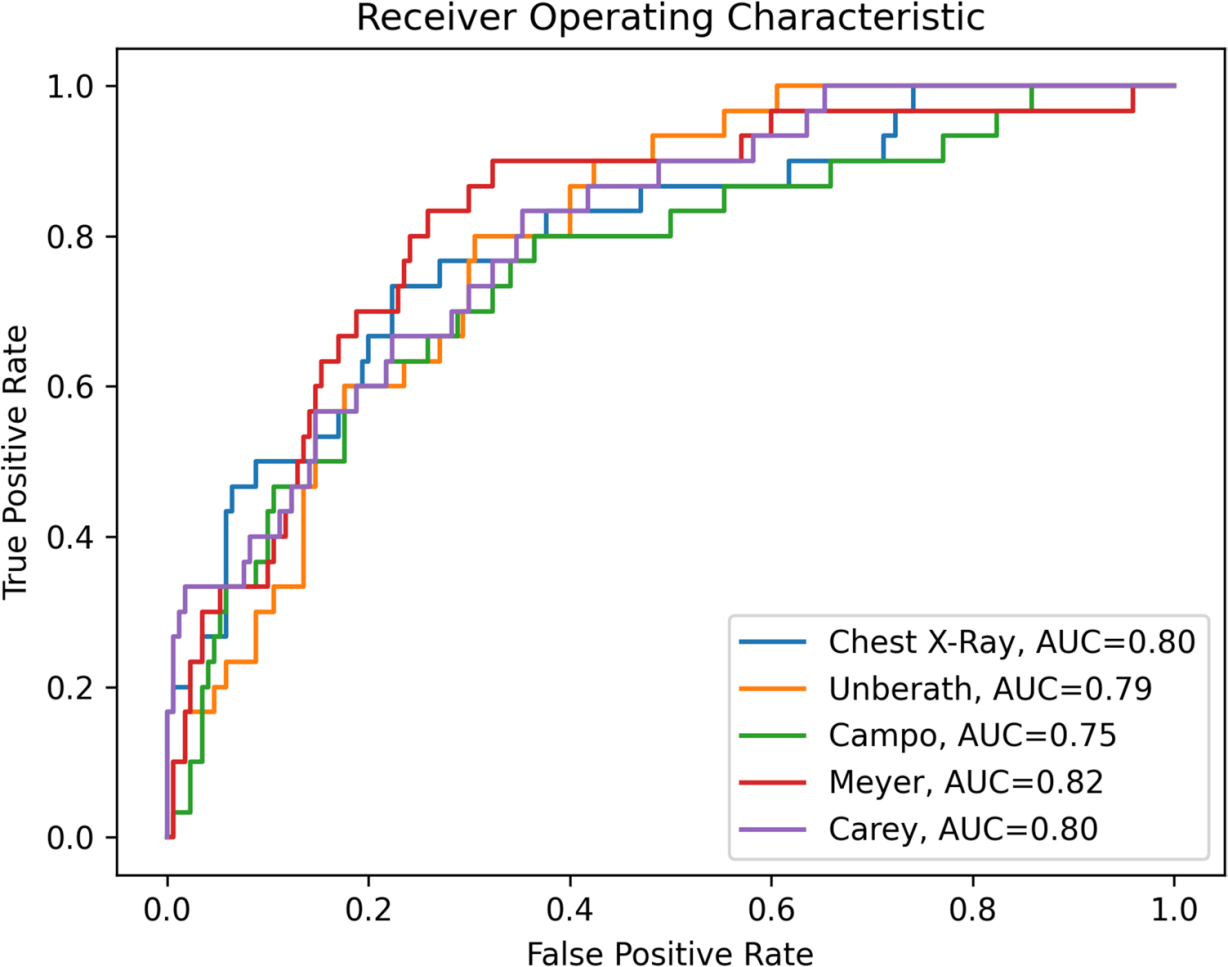
ROC curves and AUC scores for the pathology detection performance on the chest X-ray and DRRs constructed

The average AUC scores for the original CXRs and the four DRR techniques were calculated using the complete dataset (*n* = 197), as shown in Fig. 4. The AUC for CXRs was 0.80, while AUCs for the DRR techniques ranged from 0.75 to 0.82. These variations were not statistically different *(p* > 0.26)*.* Among the techniques evaluated, the SoftMip approach by Meyer [8] achieved the highest AUC score.

### Qualitative Evaluation

The results from the qualitative evaluation are summarised in Table 1.

**Table 1:** Average scores on the assessment of specified anatomic regions, with standard deviation, using a 6-point Likert scale in the qualitative evaluation

Anatomic region	Unberath [4]	Campo [6]	Meyer [8]	Carey [7]
Whole DRR	3.0 [2.2–3.8]	3.4 [2.4–4.3]	3.5 [2.5–4.4]	3.1 [2.1–4.2]
Soft tissue	4.0 [3.3–4.7]	4.3 [3.0–5.6]	4.4 [3.1–5.6]	4.2 [3.0–5.4]
Ossal structures	3.3 [2.4–4.1]	3.6 [2.5–4.7]	3.4 [2.1–4.7]	3.3 [2.5–4.1]
Mediastinum	3.6 [2.8–4.3]	3.9 [3.0–4.8]	3.9 [3.0–4.8]	3.7 [2.8–4.6]
Lungs	3.1 [2.3–3.9]	3.0 [1.8–4.2]	3.4 [2.6–4.2]	3.3 [2.4–4.3]

The DRR techniques received ratings ranging from slightly negative to neutral (3.0 to 3.5) concerning diagnostic image quality. No significant differences were found (*p* > 0.80).

The oral feedback provided by radiologists was analysed and categorised into overarching themes. The radiologists are referred to as R1 through R, and relevant quotes are provided to illustrate specific themes. The number of individual radiologists who reference a certain theme or comment is denoted by ‘n’. All comments have been translated from Dutch to English.

#### Resolution

All radiologists (*n* = 6) commented on the resolution of the generated DRRs. R2 noted, ‘The resolution of this DRR is more akin to a scout view from a CT scan than a CXR’. R4 expressed concern, stating, ‘I cannot be sure that I do not miss findings at this resolution’. The resolution of the DRR was often mentioned in conjunction with the question on the assessment of the lungs in the DRR (*n* = 4).

#### Noise

Four radiologists (R1, R2, R3, R6) discussed the level of noise present in the DRRs. R1 stated, ‘The level of noise in this DRR makes it difficult to assess the soft tissue’. Noise levels impacted the assessment of soft tissue and the overall DRR quality (*n* = 3). Interestingly, one radiologist (R6) found the noise beneficial, commenting, ‘The increase noise in this image in combination with the increased lucency makes this image more pleasant to read’.

#### Parallel Compared to Point-Source-Based Projections

Although the radiologists were unaware of the underlying DRR technique, all of them (*n* = 6) noted the difference between the parallel-based and point-source-based DRRs. R3 observed, ‘There seems to be a different distance between the dorsal ribs when comparing these DRRs’. R4 continued, ‘The chest wall seems to be further apart on this (i.e. point source) DRR compared to another (i.e. parallel based)’.

**Fig. 5:**
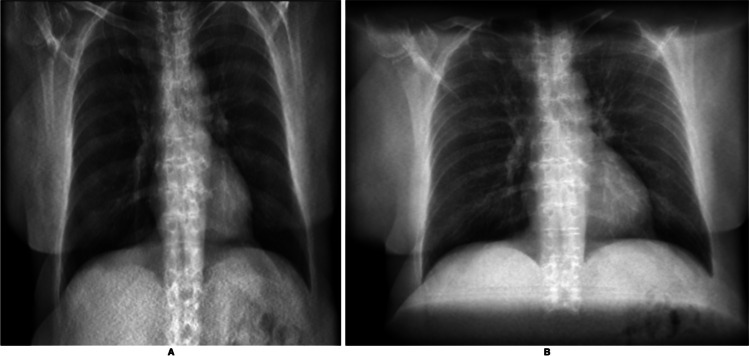
Two DRRs constructed from the same ULDCT scan. **A** DRR constructed using a parallel-based projection, **B** DRR constructed using a point-source-based projection. In the point-source-based projection, circular edges at the cranial and caudal ends obstruct the view of the anatomy

The perspective introduced by the point-source technique was cited as influencing the assessment of the DRR as a diagnostic CXR (*n* = 3), the ossal structures (*n* = 3), and the lungs (*n* = 2). In two other cases, radiologists (*n* = 3) noted that the over-projection at the cranial and caudal ends of point-source DRRs detracted from image quality. Figure 5 illustrates this effect.

#### Potential Diagnostic Application

All radiologists (*n* = 6) discussed the potential diagnostic application of DRRs. R2 commented, ‘In cases like this one, where there are no abnormalities and the inspiration is decent, the DRR is fine’. R3 added, ‘If we can improve the resolution and look-and-feel I believe the DRR would be quite close to a CXR’. The potential for DRRs to serve as equivalent and/or replacement for CXRs was conditioned on the resolution or look-and-feel of the DRR (*n* = 4).

## Discussion

In this pilot study, we compared four DRR techniques to explore which best approaches the diagnostic image quality of CXRs. No significant differences were observed in CheXNet performance between DRRs and CXRs (0.75–0.82 vs 0.80 AUC, *p* > 0.25). This aligns with previous findings by Mortani Barbosa et al. [14], who demonstrated DRRs to be non-inferior to CXRs for COVID-19 disease classification in 86 patients, suggesting that this non-inferiority may extend to other disease classes.

While the quantitative analysis using CheXNet is not novel in isolation, it serves as an important benchmark for situating our results within the broader body of DRR research. Unlike prior studies, we extend this analysis by focusing on DRRs generated from ULDCT scans and complementing it with qualitative evaluations by radiologists. Radiologists in the qualitative evaluation expressed uncertainty about the diagnostic image quality of the DRRs, rating them as neutral (3.0 to 3.5) compared to CXRs. Key differences in resolution, noise, and overall look-and-feel were noted across DRRs and between DRRs and CXRs, which may impact both AI model performance and human interpretation. In the following discussion, we explore the specific issues raised during the qualitative evaluation, highlighting areas for improvement to better align DRRs with clinical expectations.

### Image Quality Factors: CXR Versus DRR

First, the different DRR techniques use varied *attenuation* calculations, affecting pixel values and contributing to the look-and-feel of the DRR. Techniques optimised for specific applications, such as emphysema quantification or pelvis image registration, may not perform optimally in general chest imaging contexts. For example, methods focused on lung visualisations might underrepresent other anatomical regions. Conversely, the SoftMip technique, despite not being originally developed for chest imaging, showed the highest performance in our evaluations. This is potentially due to its design for ULDCT data, which aligns well with our study conditions.

Second, DRR *resolution* is inherently limited by the corresponding CT scan’s resolution. DRRs constructed from a standard 512 × 512 grid result in a resolution of 512 × Z, where Z, typically ranging from 300 to 400, represents the number of reconstructed slices. In contrast, CXRs are acquired at a resolution exceeding 3000 × 3000 pixels. All radiologists noted the higher resolution of CXRs favourably. However, this resolution disparity did not impact the performance of CheXNet, as the model down-samples all input images to 224 × 224 pixels, effectively neutralizing the difference in the original resolution between DRRs and CXRs. Nonetheless, applying established super-resolution algorithms [15] to either the DRR or the source CT images could boost radiologists’ perception of DRRs.

Third, *noise levels* in DRRs, particularly in soft tissue regions, were noted by most radiologists. In CXRs, the level of noise in this anatomic region is inherently lower compared to ULDCT-derived DRRs. Given that no pathology was present in these regions within the dataset, we could not assess the impact on CheXNet performance. While the SoftMip technique was designed to address the high noise levels in ULDCT datasets, this study did not quantify noise levels. To mitigate noise in DRRs, de-noising networks [16] can be applied to the ULDCT data, or to the DRRs itself. Alternatively, using regular dose CT data to construct DRRs may reduce noise levels.

Fourth, the *geometry* of the DRR technique affects DRR perception. Radiologists viewed point-source projections less favourably due to issues with over-projection and distortion, particularly at the caudal and cranial edges of the DRR. These edges tend to project over the central DRR, as shown in Fig. 4, obscuring the view of anatomical structures at the extremes of the image. This issue is not encountered in conventional CXRs.

Finally, variations in *window-level* settings and contrast enhancements were investigated to optimise DRR appearance but did not yield significant insights into CheXNet performance. Consequently, these variations were not included in our final analysis. Further studies could explore the effects of these adjustments on both machine learning performance and radiologists’ evaluations.

### Clinical Relevance

DRRs can be generated within seconds using standard CPU resources, resulting in four DRRs in under a second on a regular clinical workstation. Furthermore, DRRs can be derived from CT images regardless of anatomical region, contrast use, or other imaging parameters, and can be produced from any viewing angle, extending beyond the standard CXR views. Additionally, DRRs can be freely added to standard DICOM image series, serving as a versatile tool with potential value in both acquisition and diagnostic workflows. Further research is necessary to identify the specific workflows that could benefit from DRRs, determine optimal presentation methods, and assess their clinical value.

### Limitations

Our study has several limitations. The size of the dataset, where both an ULDCT and a (same-day) CXR are available for the same patient, is small, limiting the sample size available for the quantitative evaluation. Consequently, AUC scores were calculated for as few as ten cases for specific disease classes, which may limit the generalizability of these results. In this pilot study, a formal power analysis was not performed. Larger studies with diverse pathologies are necessary to confirm these findings. Additionally, the CheXNet model was trained on a dataset known to contain labelling inconsistencies, which could have affected performance [17]. In the qualitative evaluation, only DRRs without pathology were assessed, and it is possible that the presence of findings could have influenced radiologist’s opinions. On average, radiologists expressed neutral opinions regarding the diagnostic image quality of the DRRs, but the underlying motivations of a neutral rating were not surveyed. The study would have benefitted from a more structured survey to better understand the radiologist’s indecision about the diagnostic image quality of the DRRs.

In this study, DRRs were compared to CXRs rather than the source CT data because DRRs were intended to simulate the diagnostic utility of CXRs for 2D imaging applications. Comparing DRRs to CTs, which offer 3D volumetric data, would not align with this clinical use case. This does, however, represent a limitation of our study, as DRR utility relative to CT data has not been evaluated.

## Conclusion

DRRs showed comparable disease detection performance to CXRs, suggesting non-inferiority. However, radiologists expressed concerns about DRR image quality, particularly in terms of resolution, noise, and overall look-and-feel. Addressing these limitations with advanced techniques may further align DRRs with the diagnostic standards of CXRs.

## Data Availability

The data used in this study will not be made available publicly due to privacy concerns.
